# Feeder cells treated with ethanol can be used to maintain self‐renewal and pluripotency of human pluripotent stem cells

**DOI:** 10.1002/2211-5463.13538

**Published:** 2023-01-04

**Authors:** Yahui Ren, Sijin Zhang, Yun Liang, Zichao Gong, Yongyi Cui, Wei Song

**Affiliations:** ^1^ School of Life Science and Engineering Henan University of Urban Construction Pingdingshan China

**Keywords:** ethanol, feeder cells, human embryonic stem cells, human pluripotent stem cells, self‐renewal

## Abstract

Feeder cells play an important role in the culture of human pluripotent stem cells (hPSCs) *in vitro*. Previously, we used methanol as a fixative to prepare feeder cells for the cultivation of pluripotent stem cells (PSCs), and this method could maintain the self‐renewal and pluripotency of PSCs. However, methanol is toxic, and so here we examined whether ethanol could be used to prepare feeder cells as a fixative for hPSC culturing. Primed, naïve, and extended human embryonic stem cells and induced pluripotent stem cells can maintain self‐renewal and undifferentiated potential on feeder cells treated with ethanol for an extended period. RNA sequencing analysis showed that the expression of collagen‐related genes in hPSCs cultured on feeder cells treated with ethanol was significantly lower as compared with hPSCs cultured on feeder cells treated with mitomycin C. Therefore, we speculate that the signaling pathway mediated by collagen‐related genes may, at least in part, contribute to the maintenance of self‐renewal and pluripotency of PSCs induced by feeder cells treated with chemicals.

## Graphical abstract

Human fibroblasts can be used as feeder cells after treatment with ethanol. Primed, naïve and extended hPSCs can maintain the self‐renewal and undifferentiated potential on feeder cells treated with ethanol. Collagen‐related genes in pluripotent stem cells cultured on feeder cells treated with ethanol may contribute to the maintenance of the pluripotent state of pluripotent stem cells.

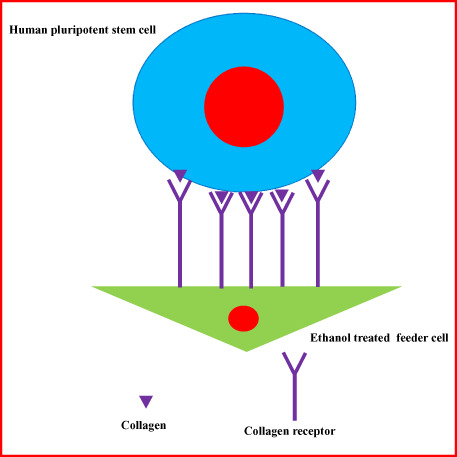

**Abbreviations**
AP: alkaline phosphatase
EBs: embryoid bodies
ECM: extracellular matrix
ESCs: embryonic stem cells
ET‐HDF: ethanol treated HDF
ET‐MEF: ethanol treated MEF
HDFs: human dermal fibroblasts
hPSCs: human pluripotent stem cells
iPSCs: induced pluripotent stem cells
LIF: leukemia inhibitory factor
MEFs: mouse embryonic fibroblasts
MMC: mitomycin c
MMC‐HDF: mitomycin c treated HDF
MMC‐MEF: mitomycin c treated MEF
PSCs: pluripotent stem cells
qRT‐PCR: quantitative reverse transcription‐polymerase chain reaction

Human pluripotent stem cells (hPSCs) have the capacity of self‐renewal and the ability to differentiate into all cell types *in vitro* and *in vivo* [1]. These characteristics instill them with an enormous promise in regenerative medicine, where they could be used in cell, tissue, and even organ‐based replacement therapy [2]. Since the establishment of the first human embryonic stem cell line in 1998, the *in vitro* culture system of hPSCs has been formally established [3]. With the development of a culture system, hPSCs are divided into several different states, including primed, naïve, and extended state. Primed hPSCs are flat in shape and rely on the Activin and Nodal signaling pathways to maintain their self‐renewal and multidirectional differentiation ability, but these state cells cannot achieve animal chimerism *in vivo* [4]. In recent years, by adding leukemia inhibitory factor (LIF) and small molecular compounds to the culture system, the primed hPSCs could be transformed into the naïve state [5]. These cells are spherical in shape and can be chimeric in mice and pigs [6]. Compared with the primed and naïve stem cells, the extended hPSCs have the potential to form the extraembryonic tissues, such as placenta and yolk sac [7].

Since then, the establishing of both the first human induced and extended pluripotent stem cell lines needs the support of feeder cells [7, 8]. The most commonly used feeder layer cells are mouse fetal fibroblasts treated with mitomycin C. Mitomycin C‐treated cells lost their ability to divide and proliferate, but the ability of secret of growth factors and cytokines, as well as their special structural characteristics, could maintain the self‐renewal ability of hPSCs [9]. The preparation of feeder cells by mitomycin C requires the isolation, culture, treatment, and cryopreservation of mouse embryonic fibroblasts (MEFs). The whole process is complex and time‐consuming, and the probability of contamination still exists [10]. In addition, hPSCs cultured on mitomycin C‐treated MEF cells, because of MEF cells are exogenous, the hPSCs cannot be used in the clinic [11]. Feeder‐free culture systems were developed to replace the feeder culture systems in recent years. Culture dishes coated with Matrigel [12], recombinant collagen [13], recombinant vitronectin [14], synthetic polymers [15], hydrogel [16, 17], and 3D scaffold [18, 19] were developed and used to culture PSCs. However, these methods either use animal products that may have potential problems in transplantation applications, or need special materials.

Previously, we and our colleagues used methanol to fix feeder cells such as fibroblasts and then were used as feeder layers for maintaining the pluripotency of mouse and human PSCs [20]. However, the toxicity of methanol is still a serious problem for the further application in culturing human PSCs. In this study, we tried to use ethanol to take the place of methanol for making feeder layer cells. Meanwhile, humanized fibroblasts were treated with ethanol as human PSCs feeder layers to maintaining the state of pluripotency. Not only the primed state, but also naïve and extended human induced pluripotent stem (iPS) and ES cells could keep their normal morphology and pluripotency for long‐term passages on ethanol‐treated feeder cells.

The mechanism of feeder cells for maintaining pluripotency of pluripotent stem cells is still unclear. As previously, we demonstrated that extracellular matrix collagen‐IV and fibronectin play crucial roles to activate the signals of self‐renewal and pluripotency [20]. Extracellular matrix (ECM) is a complex network structure composed of macromolecular substances secreted by cells from inside to outside, which supports and connects tissue structure, regulates tissue genesis, and cell physiological activities [21]. The proliferation, metabolism, and self‐renewal of pluripotent stem cells are regulated by changes in the expression of ECM and its cell surface receptors [22]. It has been reported that purified collagen [23] or fibronectin [24] serve as a matrix that can effectively maintain the undifferentiated characteristics of hPSCs.

## Materials and methods

### Cell sources

Mouse embryonic fibroblast and hES cells H1 came from the Shaanxi stem cell engineering center and were amplified and preserved by our laboratory. HDF and hiPS cells were purchased from Shanghai SiDanSai Biotechnology Co., Ltd (Shanghai, China) and were amplified and preserved in our laboratory.

### Preparation of MMC‐MEF feeder cells

To prepare MEF feeders, cells were cultured in a culture dish with MEF medium (DMEM high glucose plus 15% FBS) to 80–90% confluence and then 10 μg·mL^−1^ mitomycin C were added. After 2.5 h incubation at 37 °C, mitomycin C was removed and cells were washed with PBS three times. Cells were digested with 0.25% Trypsin–EDTA and then used immediately or stored in liquid nitrogen.

### Human dermal fibroblasts (HDFs) treated with ethanol

To prepare ethanol‐fixed feeder cells, HDFs were cultured with HDF medium (DMEM high glucose plus 15% FBS). When cells were in 80–90% confluence, media were removed and cells were washed with PBS once. The 100% ethanol precooled in 4 °C was added into the dish for 5 min at room temperature. After removing ethanol, the dish was opened and placed on the surface of flow hood clean bench for 5 min to make sure that ethanol was fully volatilized, and the fixed cells were dehydrated. Cells treated by ethanol were ready to be used as feeders immediately or stored at room temperature for future use.

### Human pluripotent stem cells culture

The primed hiPSCs and H1 were seeded on ethanol‐fixed HDF cells and cultured with DMEM/F12 (11330‐032; Thermo Fisher Scientific, Shanghai, China) supplemented with 20% knockout serum replacement (A3181502; Thermo Fisher Scientific), 1% GlutaMAX (35050‐061; Thermo Fisher Scientific), 1% NEAA (11140‐050; Thermo Fisher Scientific), 0.1 mm b‐mercaptoethanol (21985‐023; Thermo Fisher Scientific) and 10 ng·mL^−1^ bFGF (13256029; Thermo Fisher Scientific) or mTeSR 1 medium (#85850; STEMCELL Technologies, Shanghai, China).

The naive hiPSCs and H1 were seeded on ethanol‐fixed HDF cells and cultured with the Rudolf Jaenisch lab system [25]: 240 mL DMEM/F12 (11330‐032; Thermo Fisher Scientific), 240 mL Neurobasal (21103‐049; Thermo Fisher Scientific), 5 mL N_2_ supplement (17502‐048; Thermo Fisher Scientific), 10 mL B27 supplement (12587‐010; Thermo Fisher Scientific), 10 μg recombinant human LIF (LIF1050; Millipore, Shanghai, China), 1% GlutaMAX (35050‐061; Thermo Fisher Scientific), 1% NEAA (11140‐050; Thermo Fisher Scientific), 0.1 mm b‐mercaptoethanol (21985‐023; Thermo Fisher Scientific), 50 μg·mL^−1^ BSA (A1933; Sigma, Shanghai, China). Following small molecules and cytokines: 1 μm PD0325901 (S1036; Selleck, Shanghai, China), 1 μm IM‐12 (Enzo, Farmingdale State College, NY, USA; 1 μm), 0.5 μm SB590885 (2650; R&D Systems, Shanghai, China), 1 μm WH‐4‐023 (H620061; A Chemtek, Worcester, MA, USA), 10 μm Y‐27632 (S1049; Selleck), and 20 ng·mL^−1^ Activin A (AF‐120‐14E; Peprotech, Suzhou, China).

The extended hiPSCs and H1 were seeded on ethanol‐fixed HDF cells and cultured with the LCDM system [7]: 240 mL DMEM/F12 (11330‐032; Thermo Fisher Scientific), 240 mL Neurobasal (21103‐049; Thermo Fisher Scientific), 2.5 mL N_2_ supplement (17502‐048; Thermo Fisher Scientific), 5 mL B27 supplement (12587‐010; Thermo Fisher Scientific), 1% GlutaMAX (35050‐061; Thermo Fisher Scientific), 1% NEAA (11140‐050; Thermo Fisher Scientific), 0.1 mm β‐mercaptoethanol (21985‐023; Thermo Fisher Scientific), 5% knockout serum replacement (A3181502; Thermo Fisher Scientific), 10 ng·mL^−1^ recombinant human LIF (LIF1050; Millipore), 1 μm CHIR99021 (S1263; Selleck), 2 μm (S)‐(+)‐dimethindenemaleate (1425; Tocris, Shanghai, China), and 2 μm minocycline hydrochloride (sc‐203339; Santa Cruz Biotechnology, Santa Cruz, CA, USA).

### RT‐PCR

Total RNAs were extracted by TRIzol Reagent (15596026; Thermo Fisher Scientific) according to the manufacturer's instructions. RNAs were examined by measuring OD260/280 ratio, and samples with a ratio of 2.0 were used for reverse transcription (RT). One microgram of total RNAs were reverse‐transcribed using the PrimeScript RT reagent Kit (RR047A; Takara, Beijing, China). RT‐polymerase chain reactions (PCRs) were performed using Premix Taq (RR900A; Takara) for 30 cycles at 98 °C 10 s, 58 °C 30 s, and 72 °C 60 s. Non‐RT negative controls (RT−) were also performed to monitor nonspecific reactions, and *β‐Actin* was used as the internal control. Quantitative RT‐PCRs (qRT‐PCR) were performed using a 10‐fold dilution of cDNA with TB Green® Premix Ex Taq II (RR820A; Takara), and detected with the CFX‐96 Real‐Time PCR System (BioRad, Shanghai, China). Measurements were performed on three biological replicates and each reaction was performed in triplicate. The expression level of target gene was normalized to the expression level of *β‐Actin*. Melting curve analysis was conducted to confirm the specificity. Primers used in this study are listed in Table S1.

### Alkaline phosphatase staining

The process of alkaline phosphatase (AP) staining for pluripotent stem cells was determined according to the manufacturer's instructions (C3206; Beyotime, Beijing, China).

### Immunofluorescence

For immunofluorescence assays, cells were fixed with 4% paraformaldehyde for 15–30 min at room temperature. The fixed cells were washed twice with PBS, incubated with PBS containing 0.1% Triton X‐100 for 10 min, and washed three times with PBS. After blocking in BSA‐blotting buffer (1% BSA and 0.1% Tween 20 in PBS) for 30 min, cells were incubated in BSA‐blotting buffer with primary antibodies, including anti‐Oct4 (1 : 200, sc‐5279; Santa Cruz), in a humidified chamber at 4 °C overnight. After washing three times, cells were stained for 1 h with either anti‐mouse or anti‐rabbit secondary antibody (1 : 200; Proteintech, Wuhan, China). Nuclei were stained with DAPI (10 μg·mL^−1^) for 2–5 min. Microscopy was performed on a Leica fluorescence microscope (Leica Company, Shanghai, China).

### Flow cytometry analysis

For flow cytometry analysis, pluripotent stem cells were washed once with PBS and then detached with Accutase (Gibco, Shanghai, China). After centrifugation, cells were washed twice with PBS and resuspended in stain buffer (PBS with 2% FBS) for cell counting. Then 1 × 10^6^ cells were transferred into separate 1.5 mL Eppendorf tubes and incubated in 100 μL staining buffer supplemented with PE‐anti‐SSEA‐4 antibody (#330409; Biolegend, Beijing, China) for 30 min on ice, protected from light. Cells were washed twice and resuspended in 500 μL staining buffer and analyzed with FACS Calibur (BD Biosciences, Franklin Lakes, NJ, USA). FACS data were analyzed with flowjo software (Stanford University, Stanford, CA, USA).

### Embryoid body formation and spontaneous differentiation

Human pluripotent stem cells were cultured in a 35‐mm Petri dish through suspension culture (3 × 10^6^ cells/dish) in medium without LIF. The culture medium was replaced every 2 days. After 7 days in suspension culture, the formed embryoid bodies (EBs) were transferred to a gelatin‐coated culture dish, allowing the spontaneous differentiation for another 14 days. The cells were then used for detecting markers of the three germ layers by RT‐PCR.

### Karyotype analysis

Human pluripotent stem cells were grown in a 60‐mm culture dish with 50 μg·mL^−1^ colchicine for 45 min. The cells were then trypsinized and harvested for karyotype analysis. The procedure of karyotype analysis was routinely performed as in the previous description [26].

### RNA‐Seq

Total RNA was isolated from hiPSCs in primed and extended state using TRIzolTM Reagent (15596026; Thermo Fisher Scientific). Three independent samples were collected for RNA‐seq. The sequencing was carried on Illumina Novaseq 6000 (Illumina Company, San Diego, CA, USA). For bioinformatics analysis, raw reads containing an adapter of low quality (*Q*value ≤ 20) were removed, then mapped to the human reference genome (GRCh38) by hisat2 (version 2.1.0; University of Texas Southwestern Medical Center, Dallas, TX, USA). The resulting files were sorted using string tie (version 1.3.0; European Molecular Biology Laboratory, Heidelberg, Germany) to obtain the counts of each gene. For differential expression analysis, gene abundances were quantified by r package edger. FDR <0.05 and log2 (fold change) >1 were used as the threshold to define gene expression differences as significant. GO and KEGG analysis of the differentially expressed genes were performed using the r package clusterprofiler28. RNA‐seq data of this study can be accessed with accession ID SUB9907594 in NCBI.

### Statistical analysis

Values are presented as the mean ± SD. Statistical analyses were performed with spss (Stanford University, Stanford, CA, USA). Two‐way ANOVA was used to study the differences between grouped data, Student's *t*‐test was performed with one‐way analysis. Statistical significance was accepted at *P* < 0.05. All flow cytometry data were analyzed and generated by flowjo software.

## Results

### Low density of human fibroblasts fixed with ethanol is disadvantageous in maintaining the pluripotency of hPSCs

**Fig. 1:**
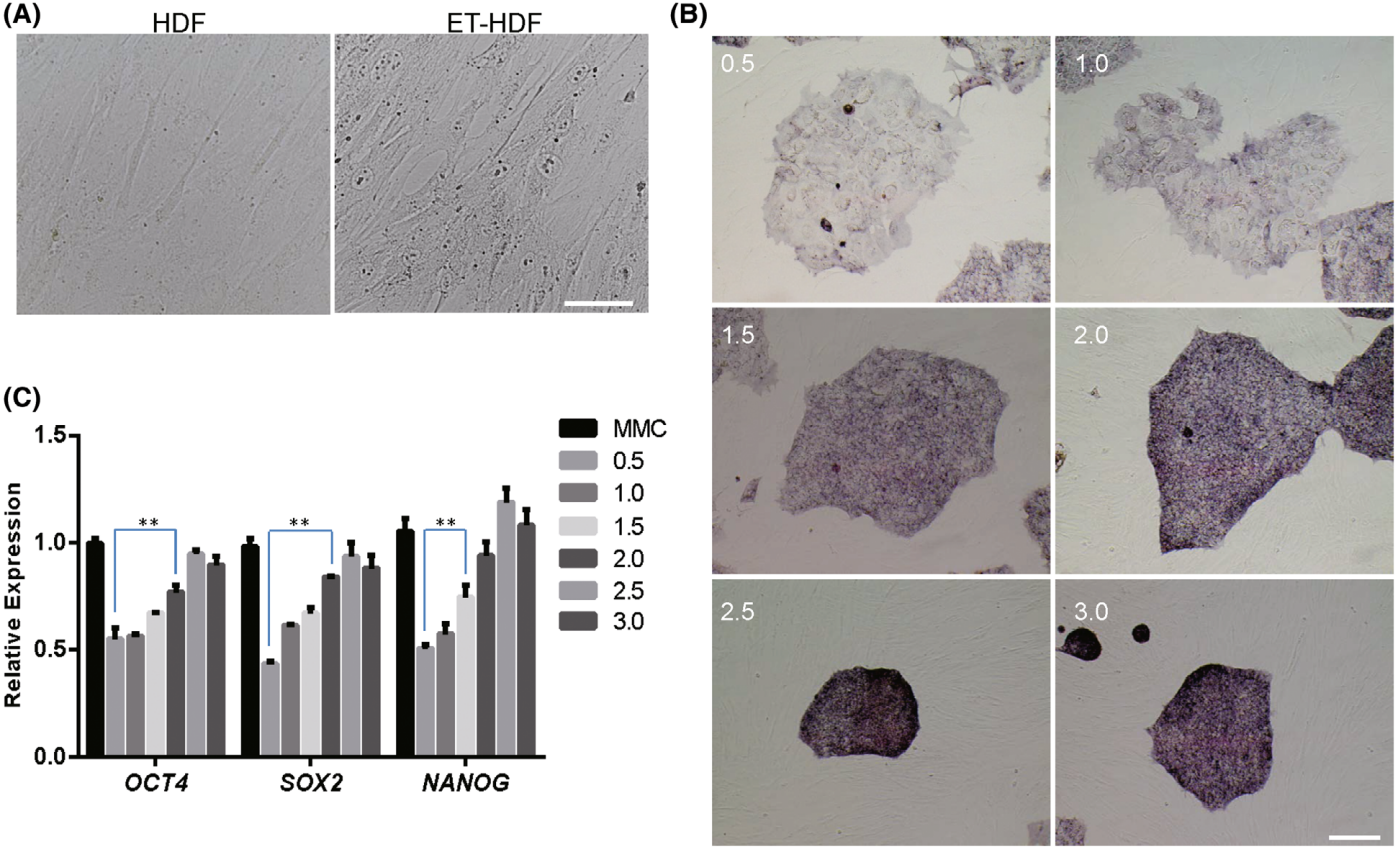
The effect of human dermal fibroblasts fixed with a different density of ethanol in culturing human iPSCs. (A) Morphology of human dermal fibroblasts fixed with ethanol (ET‐HDF). (B) AP activity of human iPSCs on human dermal fibroblasts fixed with ethanol at a different density. (C) qRT‐PCR analysis of *OCT4, SOX2*, and *NANOG* expressions in human iPSCs cultured on human dermal fibroblasts fixed with ethanol at a different density. Scale bar, 200 μm. A two‐way ANOVA statistical test was used to study the differences between the grouped data. Data indicate mean ± SD, **P < 0.01, *n* = 3.

Previous reports showed that cells fixed by methanol could be used as feeder cells to maintain mouse pluripotent stem cells [27, 28, 29]. In order to accelerate the clinical application of hPSCs, we tried to make feeder cells from human fibroblasts without exogenous substances. Because methanol is harmful to the human body, we found that ethanol can also play a role in fixing cells by optimizing the fixative, and the side effects of ethanol were small. Human dermal fibroblasts were fixed by ethanol and then attached tightly on the cell culture dishes (Fig. 1A).

Cell density of the feeder layer is very important to maintain the pluripotency of pluripotent stem cells. In order to get a better culture condition, we optimized the cell density of feeder layer. HDFs from 0.5 × 10^4^ cm^−2^ to 3.0 × 10^4^ cm^−2^ were fixed by ethanol and used to culture hiPSCs. The results showed that when the density of human fibroblasts fixed by ethanol was lower than 2.0 × 10^4^ cm^−2^, the edge of human induced pluripotent stem cells was rough and the activity of AP was low (Fig. 1B). We further detected the expression of pluripotency genes, compared with the traditional mitomycin C‐treated feeder cells; the results showed that when the density of ethanol‐fixed feeder cells was lower than 2.5 × 10^4^ cm^−2^, the expression of *OCT4*, *SOX2*, and *NANOG* was decreased significantly (Fig. 1C). In summary, the density of ethanol‐fixed fibroblasts should not be less than 2.5 × 10^4^ cm^−2^; the pluripotency of human induced pluripotent stem cells could be influenced by the low density of ethanol‐fixed feeder cells.

### hPSCs could be differentiated when cultured on a low concentration ethanol‐fixed human fibroblasts

**Fig. 2:**
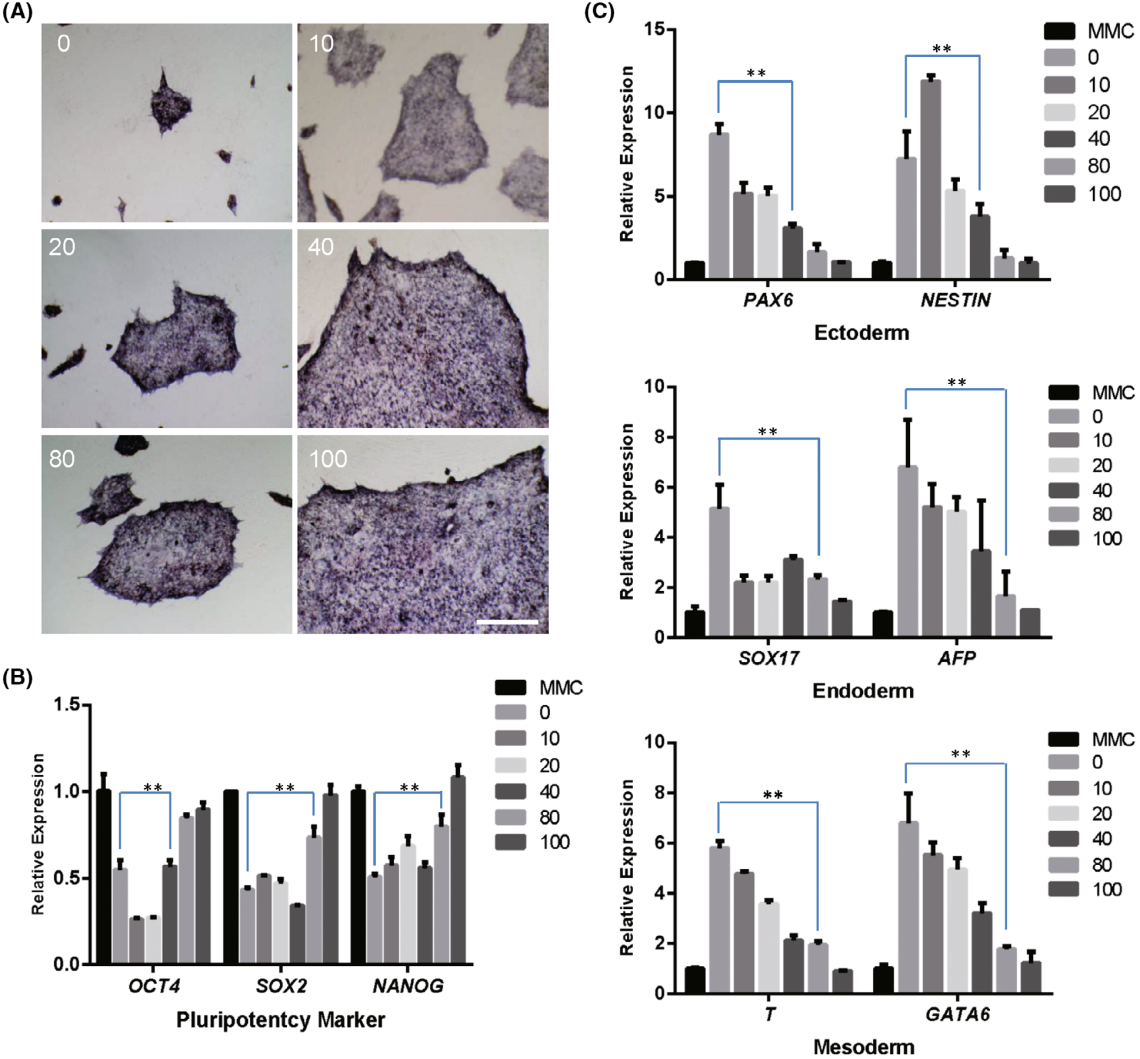
Human iPSCs cultured on human dermal fibroblasts fixed by ethanol with different concentrations. (A) AP activity of human iPSCs cultured on human dermal fibroblasts fixed with different concentrations of ethanol. (B) qRT‐PCR analysis of *Oct4*, *Sox2*, and *Nanog* expressions in human iPSCs cultured on human dermal fibroblasts fixed with different concentrations of ethanol. (C) qRT‐PCR analysis of genes of ectoderm, endoderm, and mesoderm layers expression in human iPSCs cultured on human dermal fibroblasts fixed with different concentrations of ethanol. Scale bar, 200 μm. A two‐way ANOVA statistical test was used to study differences between the grouped data. Data indicate mean ± SD, **P < 0.01, *n* = 3.

We used a different concentration of ethanol to fix HDF cells and detected the effect on the culture of hPSCs. The results of cell morphology and AP staining showed that when HDF cells were fixed by ethanol the concentration was lower 80%, the AP staining of hiPSC cells faded (Fig. 2A), and the *OCT4*, *SOX2*, and *NANOG* expression level was decreased compared with the traditional mitomycin C‐treated feeder cells (Fig. 2B).

In addition, we detected differentiated genes of hiPSC cells cultured on a different concentration ethanol‐fixed human fibroblasts; the results showed that the expression of ectoderm genes *PAX6*, *NESTIN*, endoderm genes *SOX17*, *AFP* and mesoderm genes *T*, *GATA6* was increased when the concentration of ethanol was lower than 80% (Fig. 2C). Based on this observation, a 100% concentration of ethanol was used to fix human fibroblasts.

### Maintenance of self‐renewal and pluripotency of hiPSCs on ethanol‐fixed feeder cells

**Fig. 3:**
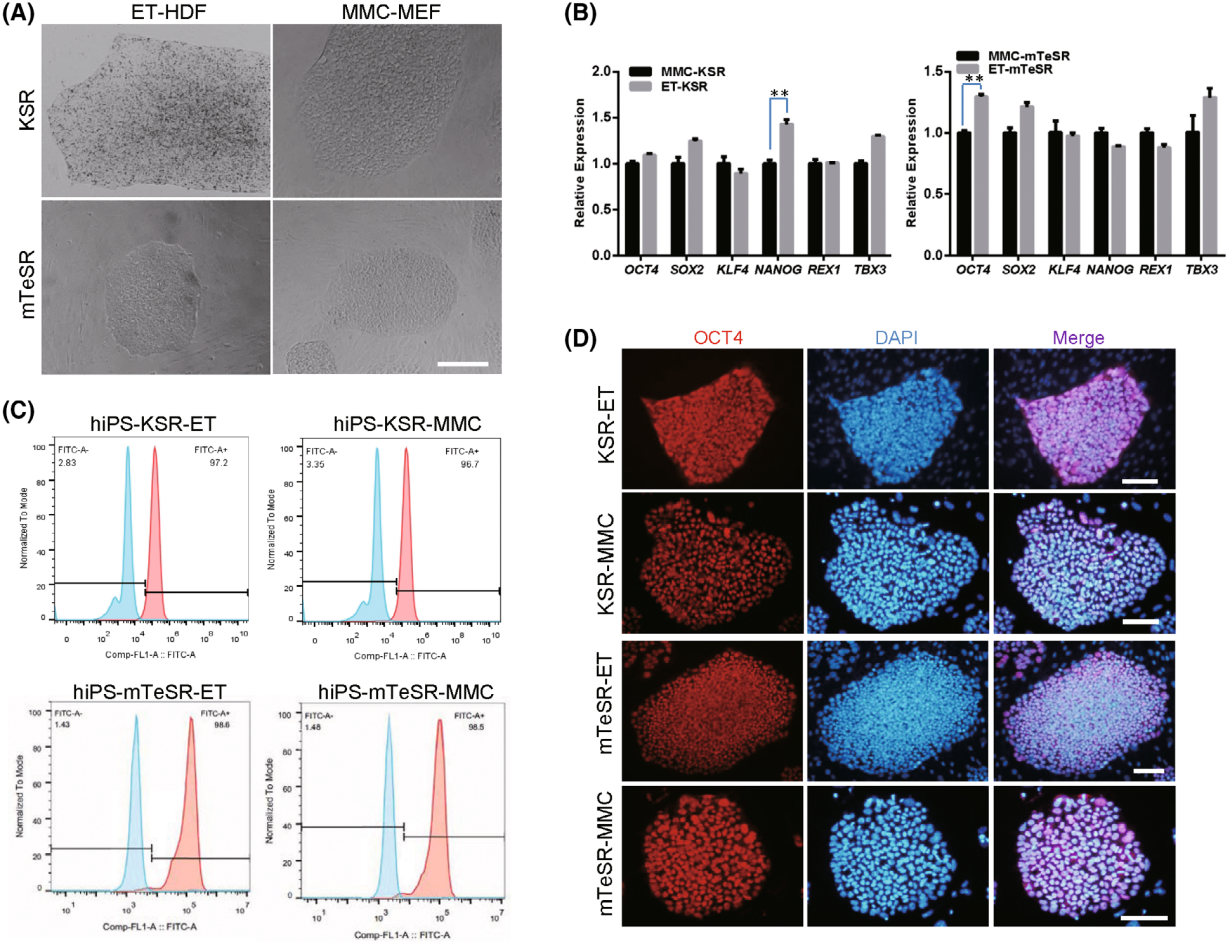
Maintenance of self‐renewal and pluripotency of primed human iPSCs on ethanol‐treated fibroblasts. (A) Primed human iPSCs were cultured on ethanol‐fixed human dermal fibroblasts (ET‐HDF). Mitomycin C‐treated MEF (MMC‐MEF) was used as the control. (B) qRT‐PCR analysis of pluripotent genes in human iPSCs cultured on ET‐HDF and MMC‐MEF feeders. (C) Flow cytometry analyses of pluripotent markers SSEA‐4 in human iPSCs cultured on ET‐HDF and MMC‐MEF feeders. (D) Immunofluorescence analyses of pluripotent markers OCT4 in human iPSCs cultured on ET‐HDF and MMC‐MEF feeders. Nuclei were stained by DAPI. Scale bar, 200 μm. The Student's *T* test was performed with one‐way analysis. Data indicate mean ± SD, **P < 0.01, *n* = 3.

**Fig. 4:**
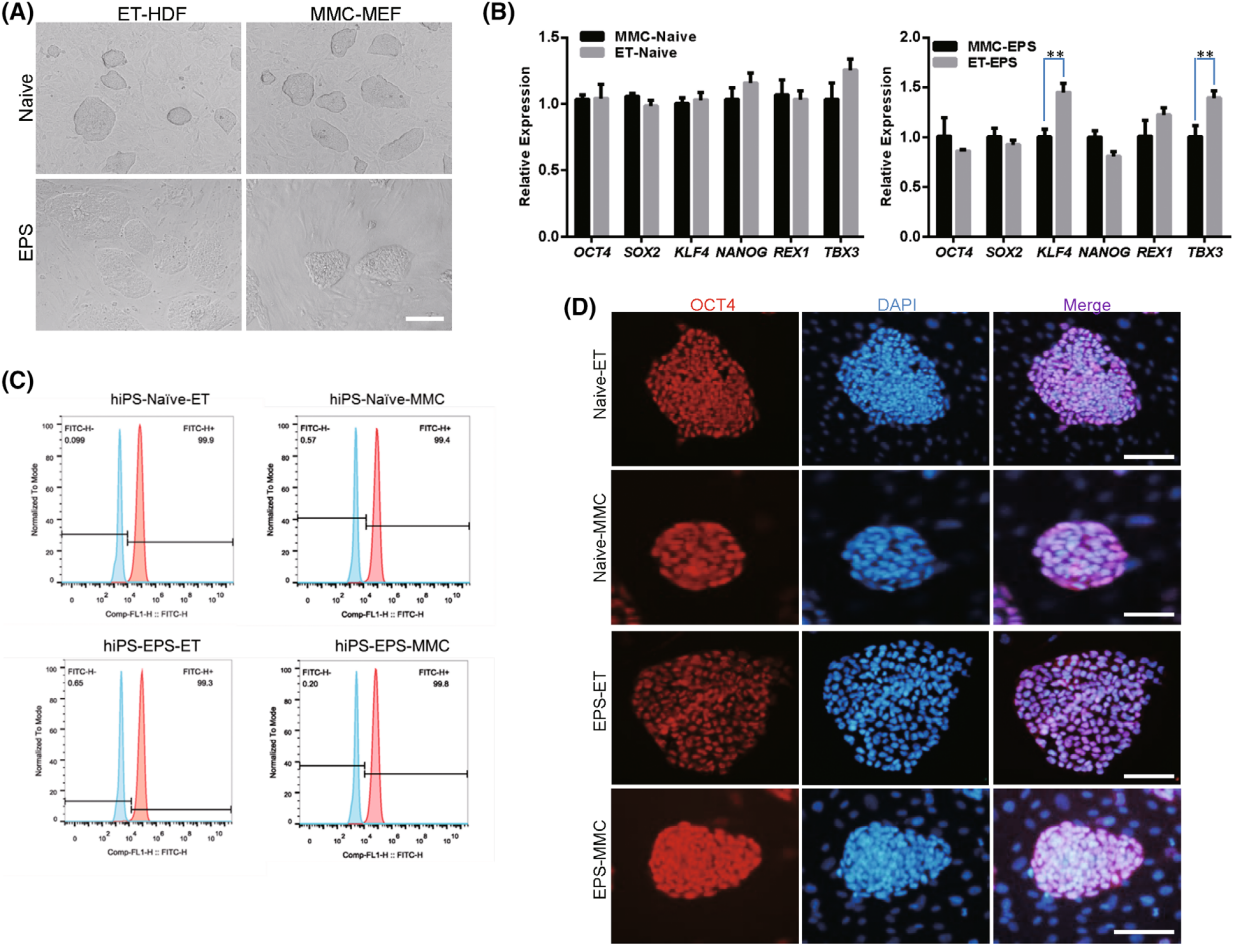
Maintenance of self‐renewal and pluripotency of naïve and extended human iPSCs on ethanol‐treated fibroblasts. (A) Naïve and extended human iPSCs were cultured on ethanol‐fixed human dermal fibroblasts (ET‐HDF). Mitomycin C‐treated MEF (MMC‐MEF) was used as a control. (B) qRT‐PCR analysis of pluripotent genes in naïve and extended human iPSCs cultured on ET‐HDF and MMC‐MEF feeders. (C) Flow cytometry analyses of pluripotent markers SSEA‐4 in naïve and extended human iPSCs cultured on ET‐HDF and MMC‐MEF feeders. (D) Immunofluorescence analyses of pluripotent markers OCT4 in naïve and extended human iPSCs cultured on ET‐HDF and MMC‐MEF feeders. Nuclei were stained by DAPI. Scale bar, 50 μm. The Student's *T* test was performed with one‐way analysis. Data indicate mean ± SD, **P < 0.01, *n* = 3.

Human induced pluripotent stem cells were seeded on ethanol‐fixed HDF (ET‐HDF) cells, ethanol‐fixed MEF (ET‐MEF) cells, mitomycin C‐treated MEF (MMC‐MEF) cells, and mitomycin C‐treated HDF (MMC‐HDF) cells. The results showed that the morphology of primed, naïve, and extended state of hiPS cells cultured on ET‐HDF was similar to that cultured on MMC‐MEF, presenting the flat and domed cell types (Figs 3A and 4A). We did qRT‐PCR analysis of pluripotent genes of primed, naïve, and extended state hPSCs that were cultured on ET‐HDF. The results indicated that the expression of *OCT4*, *SOX2*, *KLF4*, *NANOG*, *REX1*, and *TBX3* was increased versus on MMC‐MEF (Figs 3B and 4B). Positive cells for OCT4 and SSEA‐4 of hPSCs that were cultured on ET‐HDF for over 30 passages were confirmed by the flow cytometry analysis (Figs 3C and 4C) and immunofluorescence assays (Figs 3D and 4D).

We also derived the ethanol‐fixed MEF (ET‐MEF) cells and mitomycin C‐treated HDF (MMC‐HDF) cells. Human induced pluripotent stem cells were seeded on ethanol‐fixed MEF (ET‐MEF) cells, and mitomycin C‐treated HDF (MMC‐HDF) cells. The results showed that the morphology of hiPS cells cultured on ET‐MEF was similar to that cultured on MMC‐HDF, presenting the flat and domed cell types (Fig. S1A). We did qRT‐PCR analysis of pluripotent genes of primed state hPSCs that were cultured on ET‐MEF. The results indicated that expression of *OCT4*, *SOX2*, *KLF4*, *NANOG*, *REX1*, and *TBX3* was not significantly changed versus on MMC‐HDF (Fig. S1B). Positive cells for OCT4 and SSEA‐4 of hPSCs that were cultured on ET‐MEF and MMC‐HDF were confirmed by the flow cytometry analysis (Fig. S1C) and immunofluorescence assays (Fig. S1D). These results indicated that primed, naïve, and the extended state of hPSCs could maintain their self‐renewal and pluripotency on feeder cells derived from ethanol‐fixed fibroblasts in the long term.

### Growth of human ES cells H1 on ethanol‐fixed fibroblasts

**Fig. 5:**
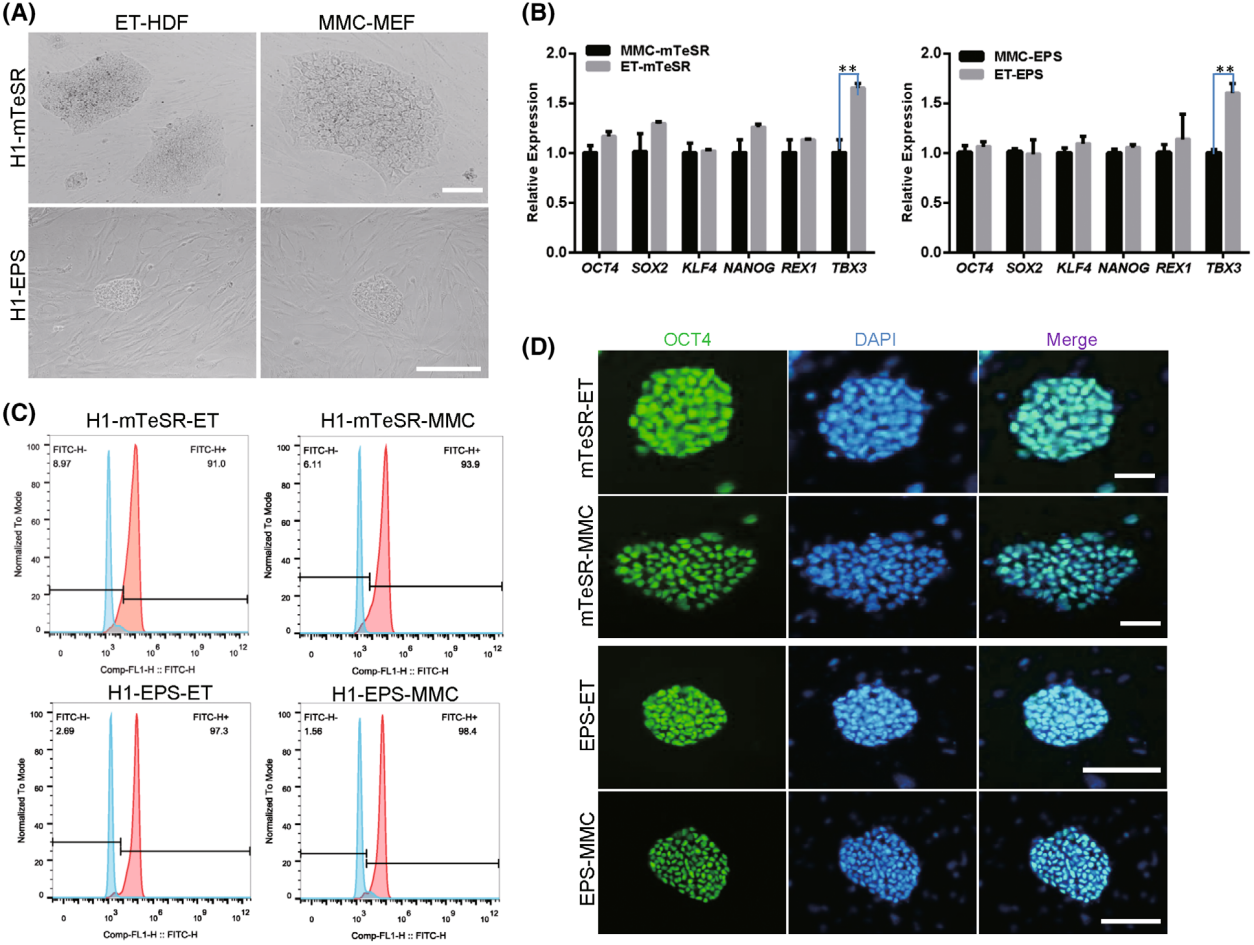
Maintenance of self‐renewal and pluripotency of human ES cells H1 on ethanol‐treated fibroblasts. (A) Naïve and extended human iPSCs were cultured on ethanol‐fixed human dermal fibroblasts (ET‐HDF). Mitomycin C‐treated MEF (MMC‐MEF) was used as the control. (B) qRT‐PCR analysis of pluripotent genes in naïve and extended human iPSCs cultured on ET‐HDF and MMC‐MEF feeders. (C) Flow cytometry analyses of pluripotent markers SSEA‐4 in naïve and extended human iPSCs cultured on ET‐HDF and MMC‐MEF feeders. (D) Immunofluorescence analyses of pluripotent markers OCT4 in naïve and extended human iPSCs cultured on ET‐HDF and MMC‐MEF feeders. Nuclei were stained by DAPI. Scale bar, 50 μm. The Student's *T* test was performed with one‐way analysis. Data indicate mean ± SD, **P < 0.01, *n* = 3.

We also used ethanol‐fixed fibroblasts to maintain human ES cells H1 cells and confirmed that primed and extended H1 cells cultured on ET‐HDF retained the typical morphology (Fig. 5A). The expression level of pluripotent genes *OCT4*, *SOX2*, *KLF4*, *NANOG*, and *REX1*of H1 was not changed compared to that on MMC‐MEF, and *TBX3* was significantly increased compared with that cultured on MMC‐MEF (Fig. 5B). Positive cells for OCT4 and SSEA‐4 of H1 cells that were cultured on ET‐HDF for 20 passages was confirmed by immunofluorescence assays (Fig. 5D) and flow cytometry analysis (Fig. 5C). These observations indicate that ethanol‐fixed fibroblasts can be used, not only to maintain human induced pluripotent stem cells, but also to maintain human embryonic pluripotent stem cells.

### Differentiation ability and karyotype analysis of hPSCs cultured on ethanol‐treated fibroblasts

**Fig. 6:**
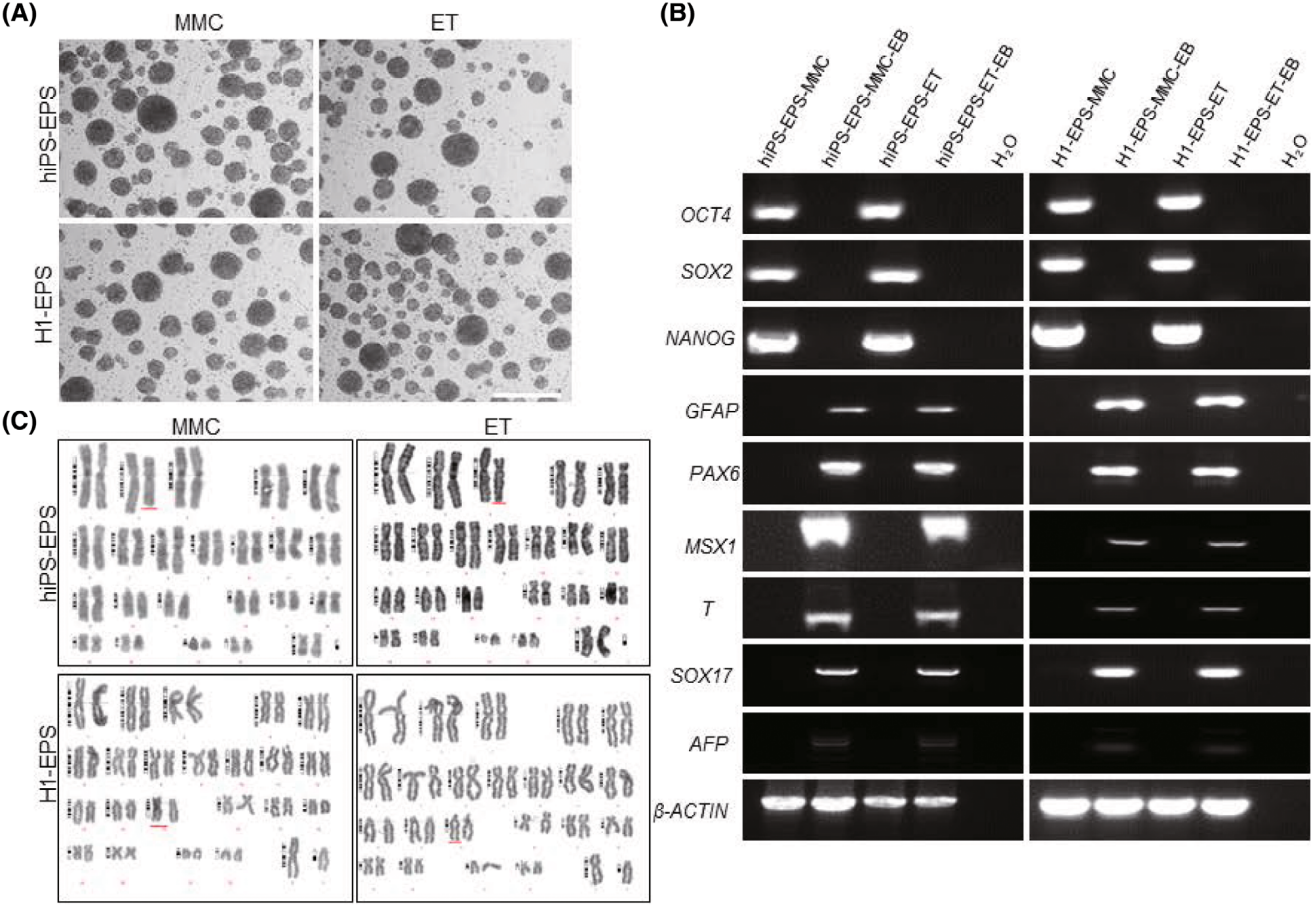
Differentiation ability and karyotype analysis of hPSCs on ethanol‐treated fibroblasts. (A) Embryoid body formation of hPSCs cultured on ET‐HDF and MMC‐MEF feeders. (B) RT‐PCR analysis of differentiation genes in the embryoid body of hPSCs cultured on ET‐HDF and MMC‐MEF feeders. (C) Karyotype analysis of hPSCs cultured on ET‐HDF and MMC‐MEF feeders. Scale bar, 100 μm.

To test the ability of the differentiation of hPSCs, we did an embryoid body formation and spontaneous differentiation experiment. The extended hiPS and H1 cells cultured on ET‐HDF could form the embryoid body in a suspension culture system (Fig. 6A). After 2 weeks adherent culture, the differentiation and pluripotent genes of three germ layers were detected by RT‐PCR. The results showed that, compared with hPSCs, the differentiation genes could be detected in the embryoid body, but the pluripotent genes could not be detected (Fig. 6B).

In order to verify the stability of the karyotype of hPSCs cultured on ethanol‐treated fibroblasts, we tested the extended state of hPSCs. The results showed that after 45 and 20 passages of extended human induced pluripotent stem cells and embryonic stem cells H1 cultured on ET‐HDF, the karyotype remained normal (Fig. 6C). The results of the differentiation ability and karyotype analysis indicated that compared with the traditional mitomycin C‐treated MEF cells, ethanol‐fixed fibroblasts could also maintain the differentiation ability and normal karyotype of hPSCs.

### RNA‐Seq analysis of the difference between hiPSCs on MMC‐MEF and ET‐HDF

**Fig. 7:**
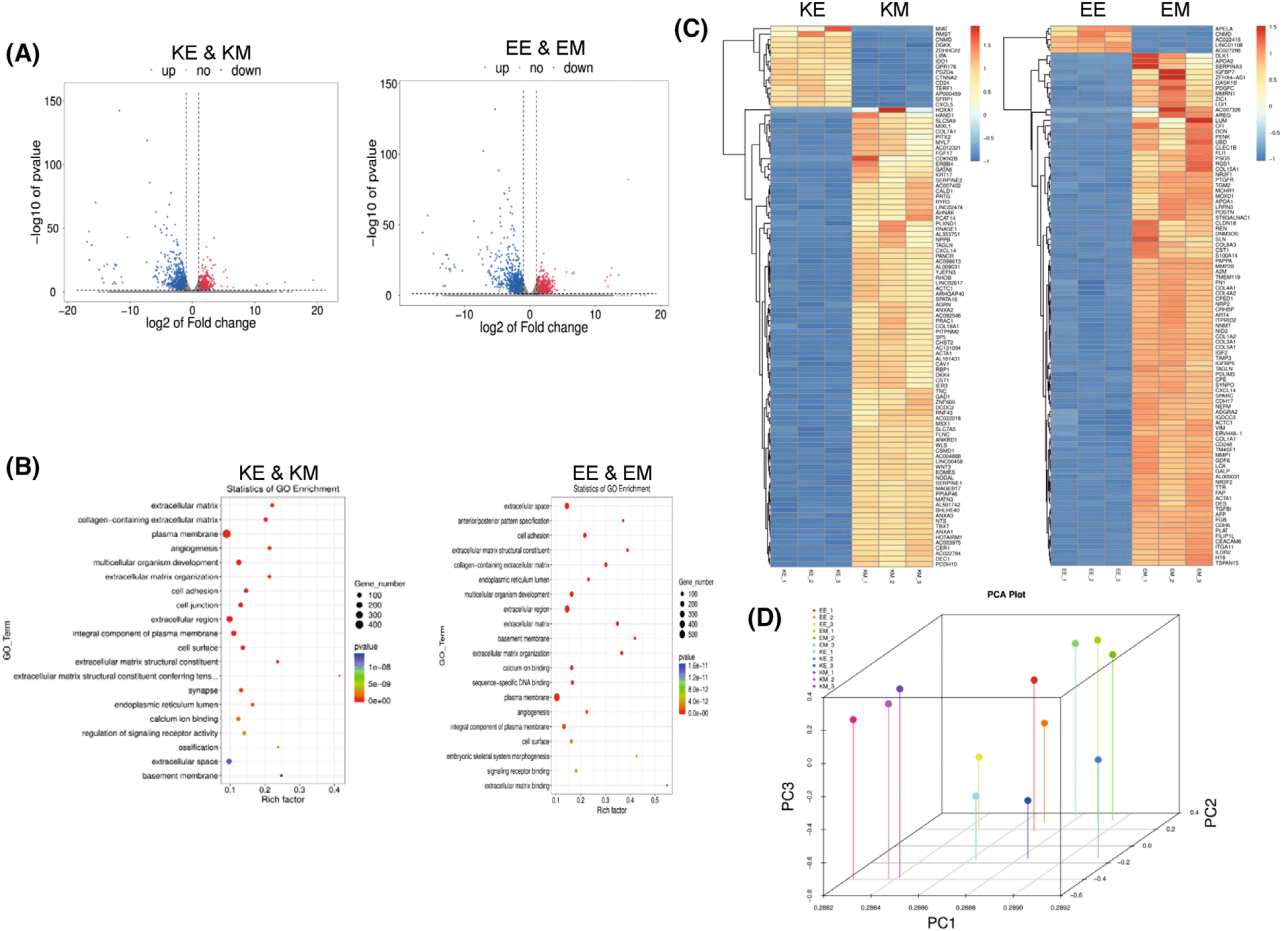
Transcriptome analysis of hiPS cells cultured on different feeder layers. (A) The volcano of different genes of primed and naive hiPS cells cultured on ethanol‐fixed HDFs versus on mitomycin C‐treated MEFs. (B) The Go enricdifferent genes of primed and naive hiPS cells cultured on ethanol‐fixed HDFs versus on mitomycin C‐treated MEFs. (C) The heatmap analysis of genes in primed and naive hiPS cells cultured on ethanol‐fixed HDFs versus on mitomycin C‐treated MEFs. (D) The principal components analysis of different genes in primed and naive hiPS cells cultured on ethanol‐fixed HDFs versus on mitomycin C‐treated MEFs. KE, the primed hiPS cells cultured on ethanol‐fixed HDFs. KM, the primed hiPS cells cultured on mitomycin C‐treated MEFs. EE, the naive hiPS cells cultured on ethanol‐fixed HDFs. EM, the naive hiPS cells cultured on mitomycin C‐treated MEFs.

In order to further explore the mechanism, we did RNA sequencing in hiPSCs cultured on MMC‐MEF and ET‐HDF. Compared with hiPSCs on MMC‐MEF, the number of genes in primed and extended hiPSCs cultured on ET‐HDF increased 619,845 and decreased 1008,1120 (Fig. 7A). The different genes in hiPSCs between the MMC‐MEF and ET‐HDF focused on cell signals about the extracellular matrix, cell adhesion and plasma membrane, etc. (Fig. 7B,C). The principal components analysis showed that there are differences in hiPSCs between MMC‐MEF and ET‐HDF (Fig. 7D).

**Fig. 8:**
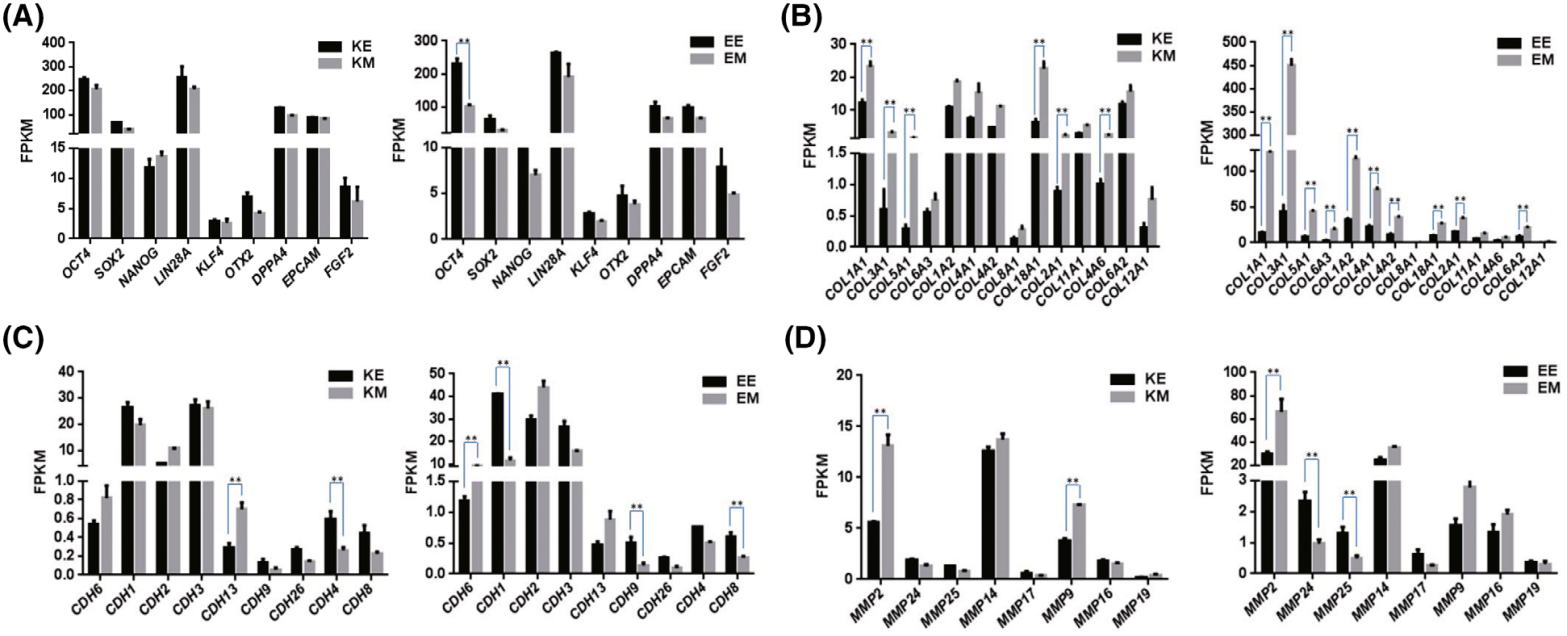
The FPKM analysis of pluripotent and cell adhesion‐related genes of hiPS cells cultured on different feeder layers. (A) The FPKM analysis of pluripotent genes of primed and naive hiPS cells cultured on ethanol‐fixed HDFs versus on mitomycin C‐treated MEFs. (B) The FPKM analysis of collagen‐related genes of primed and naive hiPS cells cultured on ethanol‐fixed HDFs versus on mitomycin C‐treated MEFs. (C) The FPKM analysis of cadherin genes in primed and naive hiPS cells cultured on ethanol‐fixed HDFs versus on mitomycin C‐treated MEFs. (D) The FPKM analysis of matrix metalloproteinase genes of different genes in primed and naive hiPS cells cultured on ethanol‐fixed HDFs versus on mitomycin C‐treated MEFs. A Student's *T* test was performed with one‐way analysis. Data indicate mean ± SD, **P < 0.01, *n* = 3.

The results of the FPKM analysis of pluripotent and cell adhesion‐related genes of hiPS cells cultured on different feeder layers demonstrated that compared with MMC‐MEF, the pluripotent genes expression of hiPSCs cultured on ET‐HDF was increased (Fig. 8A) and the collagen‐related genes was decreased (Fig. 8B). In addition, the cell adhesion and extracellular matrix genes of hiPSCs cultured on ET‐HDF changed at different degrees compared with the genes of hiPSCs cultured on MMC‐MEF (Fig. 8C,D). The collagen genes of HDF cells treated with ethanol were detected by qRT‐PCR; the results showed that the expression of collagen genes *COL1A1*, *COL3A1*, and *COL5A1* of HDF cells treated with ethanol were not significantly changed compared with HDF cells (Fig. S2).

## Discussion

Human pluripotent stem cells, including embryonic stem cells and induced pluripotent stem cells, have great potential in organ failure and cells or tissues diseases that cannot be cured by drugs or surgery in the clinic, due to their unlimited proliferation and multidirectional differentiation potential [30]. The establishment of hPSCs *in vitro* were cultured on MEF feeder cells treated with mitomycin C, including the establishment of the first human embryonic stem cell in 1998 [31] and the first human induced pluripotent stem cell in 2007 [8]. However, because of coculturing with MEF cells, this culture system can not completely eliminate the residual MEF cells, which limits the large‐scale clinical application of hPSCs. Therefore, scientists have developed feeder free culture systems for hPSCs. Various types of matrices, including Matrigel [12], recombinant collagen [13], recombinant vitronectin [14], synthetic polymers [15], hydrogel [16, 17], and 3D scaffold [18, 19], are used to support the large‐scale culture of hPSCs *in vitro*. However, these matrixes are complex, or expensive, or need special culture medium, which cannot completely solve the current situation of large‐scale cultivation of hPSCs for a clinical application.

The traditional method of feeder cells preparation is to treat MEF cells with mitomycin C or irradiation to make them lose the ability of proliferation, which is used to support the culturing of pluripotent stem cells *in vitro* [32]. However, this method needs to isolate and culture MEF cells first, which is complex and time‐consuming, and is not conducive to the large‐scale culture of pluripotent stem cells [33, 34]. In 2012, Ito and colleagues used glutaraldehyde or paraformaldehyde as the fixative to fix MEF cells and used them to culture mouse‐induced pluripotent stem cells [28]. It was found that the fixed MEF cells could replace the traditional feeder cells and maintain the pluripotent and undifferentiated state of mouse‐induced pluripotent stem cells. Subsequently, in 2015 the same team further confirmed that feeder cells fixed with glutaraldehyde or paraformaldehyde can support the *in vitro* culture of human induced pluripotent stem cells [27]. However, due to the chemical properties of glutaraldehyde and paraformaldehyde, they are not volatile and have a certain toxicity, so there may be some risks and uncertainties in large‐scale cultivation of hPSCs. In 2018, our team optimized the fixation method of glutaraldehyde or paraformaldehyde, and then used the more volatile methanol as the fixative to prepare feeder cells [20]. It was found that the methanol fixation method could perfectly replace the glutaraldehyde or paraformaldehyde method for the preparation of feeder cells. The feeder cells prepared by the methanol fixation method can be used not only for the culture of mouse pluripotent stem cells, but also for the culture of human and pig pluripotent stem cells, and can be reused and preserved for a long time [20]. In addition, Huang *et al*. [35] used ethanol‐inactivated MEF cells to culture human embryonic stem cells. The inactivated MEF cells were digested with enzyme, plated onto 0.1% gelatin‐coated dish or plate, and used freshly, or freezing and stored in liquid nitrogen for future experiments [35]. This procedure is similar to the mitomycin C method, which is also complex and time‐consuming, and it may increase the risk of hPSCs contamination when cultured on the live MEF cells inactivated by ethanol. Based on these facts, our team further screened new fixatives and found that ethanol can also be used as fixatives, and the feeder cells prepared with ethanol can support the long‐term culture of hPSCs *in vitro*. Ethanol is an ideal fixative, in which the ethanol fixation method can be used as one of the methods for the large‐scale culture of hPSCs.

The culture of pluripotent stem cells *in vitro* is inseparable from the support of feeder cells. Feeder cells can support pluripotent stem cells in many respects, but the specific molecular mechanism is still unclear. First, the substances secreted by feeder cells, including cytokines, growth factors, and immune factors, may play an important role in the self‐renewal and pluripotency maintenance of pluripotent stem cells [11]. Second, the cell adhesion signaling pathway mediated by feeder cells membrane‐associated proteins has a certain effect on the proliferation and self‐renewal of pluripotent stem cells [13]. In addition, the topology of feeder cells may also affect the proliferation of pluripotent stem cells [9]. The feeder cells prepared by chemical fixation lost the ability of secreting related substances; however, the pluripotent stem cells can still be cultured on it for a long time and maintain their pluripotency. This fact indicates that the related substances secreted by feeder cells may not be necessary for the growth of pluripotent stem cells. It can be further speculated that the feeder cell membrane proteins and their topological structure may be the key factors affecting the proliferation, self‐renewal, and pluripotency of pluripotent stem cells. Based on this, we sequenced the transcriptome of human induced pluripotent stem cells cultured on the feeder cells prepared by mitomycin C and ethanol fixation. Through analysis, we found that the expression of collagen‐related genes of human induced pluripotent stem cells cultured on the feeder cells prepared by ethanol fixation was higher than that of the feeder cells prepared by mitomycin C. There was no significant difference in the expression of genes related to pluripotency in hPSCs between culturing on mitomycin C and ethanol fixation feeder cells. Therefore, we speculate that ethanol‐fixed feeder cells affect the expression of collagen‐related proteins in hPSCs. We need to further explore the ethanol‐fixed feeder cells through which proteins and the signaling pathway affect the expression of collagen‐related proteins in hPSCs, and what the effects of decreased collagen expression are on pluripotent stem cells.

## Conclusion

In this study we optimized the method of chemical fix feeder cells and found that ethanol replacing methanol as a new fixer was practicable and effective. Human dermal fibroblasts were cultured at 80–90% convergence degree in dishes, and then removing the medium, after washing by PBS, and adding 100% ethanol for 3 min. The ethanol‐fixed feeder cells could be used after removing the ethanol and drying in clean benches for 5 min. Not only in the primed state, but also naïve and extended human iPS and ES cells could keep their normal morphology, pluripotency, and undifferentiated state for long‐term passages on the ethanol‐fixed humanized fibroblasts. RNA‐Seq analysis revealed that compared with the human PSCs cultured on MMC‐MEF, the collagen‐related genes expression of human PSCs cultured on ethanol‐fixed feeder cells decreased significantly; meanwhile, the pluripotent genes increased slightly. We hypothesized that collagen‐related genes may play important roles in human PSCs adhesion and self‐renewal through signals from feeder cells.

## Conflict of interest

The authors declare no conflict of interest.

## Author contributions

WS and YR conceived and designed the experiments. YR, SZ, YL, and YC performed the experiments. YR and WS analyzed the data. YR and ZG contributed reagents/materials/analysis tools. YR and WS wrote the article. All authors have given approval to the final version of the article.

## Supporting information


**Fig. S1.** Maintenance of self‐renewal and pluripotency of human iPSCs on ethanol treated MEF and mitomycin C treated HDF.
**Fig. S2.** Analysis of the collagen genes of HDF cells treated with ethanol.
**Table S1.** Primers used in this study.

## Data Availability

The data that support the findings of this study are available on request from the corresponding author.
