# Supplementary material for: Feeder cells treated with ethanol can be used to maintain self‐renewal and pluripotency of human pluripotent stem cells
Source: FEBS Open Bio. 2023 Jan 4;13(2):279–92. doi: 10.1002/2211-5463.13538 (PMC9900095; doi:10.1002/2211-5463.13538)
Supplement: Supplementary file 1 — Fig. S1. Maintenance of self‐renewal and pluripotency of human iPSCs on ethanol treated MEF and mitomycin C treated HDF. Fig. S2. Analysis of the collagen genes of HDF cells treated with ethanol. Table S1. Primers used in this study. [file FEB4-13-279-s001.docx]

Table S1 Primers used in this study

| Name | Sequence (5' - 3') |
| --- | --- |
| h-q-*OCT4* | F: GCTCGAGAAGGATGTGGTCC |
|  | R: CGTTGTGCATAGTCGCTGCT |
| h-q-*SOX2* | F: GCCGAGTGGAAACTTTTGTCG |
|  | R: GGCAGCGTGTACTTATCCTTCT |
| h-q-*NANOG* | F: TTTGTGGGCCTGAAGAAAACT |
|  | R: AGGGCTGTCCTGAATAAGCAG |
| h-q-*KLF4* | F: CAGCTTCACCTATCCGATCCG |
|  | R: GACTCCCTGCCATAGAGGAGG |
| h-q-*REX1* | F: GGAATGTGGGAAAGCGTTCGT |
|  | R: CCGTGTGGATGCGCACGT |
| h-q-*TBX3* | F:GAGGCTAAAGAACTTTGGGATCA |
|  | R: CATTTCGGGGTCGGCCTTA |
| h-q-*PAX6* | F: CTTTGCTTGGGAAATCCGAG |
|  | R: AGCCAGGTTGCGAAGAACTC |
| h-q-*NESTIN* | F: TTGCAGACACCTGGAAGAAG |
|  | R: GGGGAAGAGAAGGATGTTGG |
| h-q-*SOX17* | F: GAGCCAAGGGCGAGTCCCGTA |
|  | R: CCTTCCACGACTTGCCCAGCAT |
| h-q-*AFP* | F: AGCAGCTTGGTGGTGGATGA |
|  | R: CCTGAGCTTGGCACAGATCCT |
| h-q-*T* | F: ACCCAGTTCATAGCGGTGAC |
|  | R: CCATTGGGAGTACCCAGGTT |
| h-q-*GATA6* | F: CCCACAACACAACCTACAGC |
|  | R: GCGAGACTGACGCCTATGTA |
| h-q-β-*ACTIN* | F: ATAGCAACGTACATGGCTGG |
|  | R: CACCTTCTACAATGAGCTGC |
| h-RT-*OCT4* | F: GACAGGGGGAGGGGAGGAGCTAGG |
|  | R: CTTCCCTCCAACCAGTTGCCCCAAAC |
| h-RT-*SOX2* | F: GGGAAATGGGAGGGGTGCAAA AGAGG |
|  | R: TTGCGTGAGTGTGGATGGGATTGGTG |
| h-RT-*NANOG* | F: CAGCCCCGATTCTTCCACCAGTCCC |
|  | R: CGGAAGATTCCCAGTCGGGTTCACC |
| h-RT-*GFAP* | F: GGCCCGCCACTTGCAGGAGTACCAGG |
|  | R: CTTCTGCTCGGGCCCCTCATGAGACG |
| h-RT-*PAX6* | F: ACCCATTATCCAGATGTGTTTGCCCGAG |
|  | R: ATGGTGAAGCTGGGCATAGGCGGCAG |
| h-RT-*MSX1* | F: CGAGAGGACCCCGTGGATGCAGAG |
|  | R: GGCGGCCATCTTCAGCTTCTCCAG |
| h-RT-*T* | F: GCCCTCTCCCTCCCCTCCACGCACAG |
|  | R: CGGCGCCGTTGCTCACAG ACCACAGG |
| h-RT-*SOX17* | F: CGCTTTCATGGTGTGGGCTAAGGACG |
|  | R: TAGTTGGGGTGGTCCTGCATGTGCTG |
| h-RT-*AFP* | F: GAATGCTGCAAACTGACCACGCTGGAAC |
|  | R: TGGCATTCAAGAGGGTTTTCAGTCTGGA |
| h-RT-*β*-*ACTIN* | F: ATCTGGCACCACACCTTCTACAATGAGCTGCG |
|  | R: CGTCATTACTCCTGCTTGCTDATCCACATCTGC |

h, human; q, q-RT-PCR; RT, RT-PCR

Figure S1. Maintenance of self-renewal and pluripotency of human iPSCs on ethanol treated MEF and Mitomycin C treated HDF.


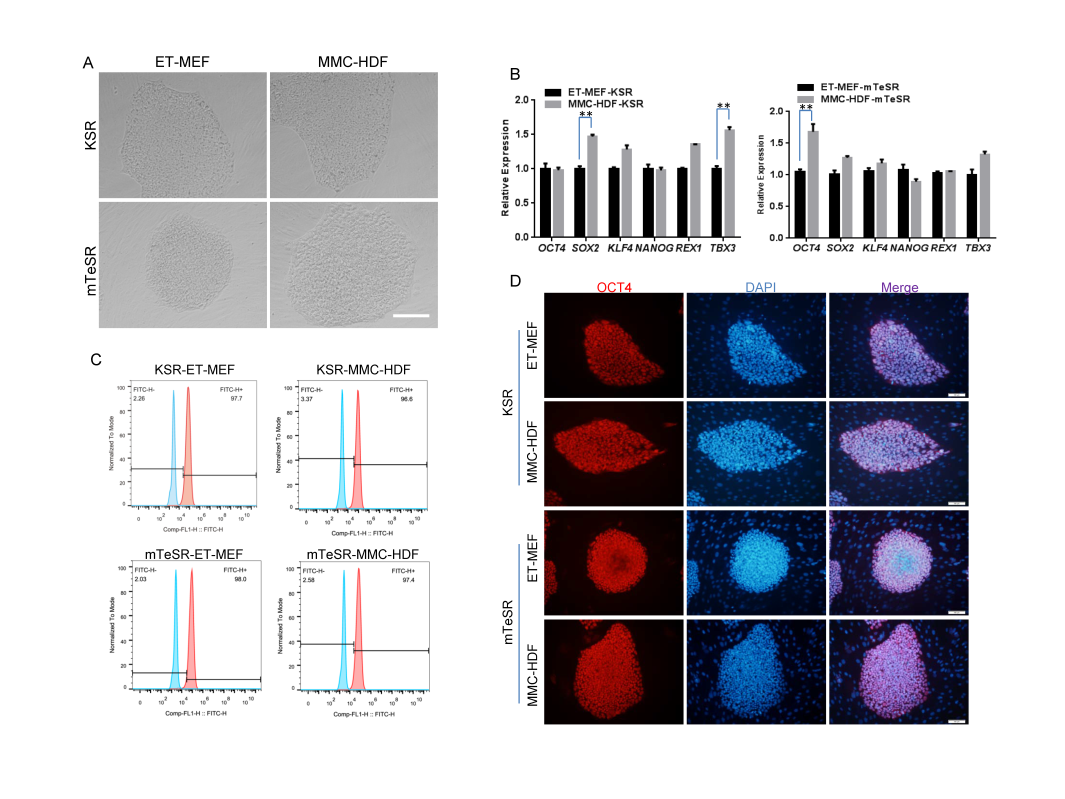


Figure S1. Maintenance of self-renewal and pluripotency of primed human iPSCs on ethanol treated MEF and Mitomycin C treated HDF. (A) Primed human iPSCs were cultured on ethanol fixed MEF (ET-MEF) and Mitomycin C treated HDF (MMC-HDF). (B) qRT-PCR analysis of pluripotent genes in human iPSCs cultured on ET-MEF and MMC-HDF feeders. (C) Flow cytometry analyses of pluripotent markers SSEA-4 in human iPSCs cultured on ET-MEF and MMC-HDF feeders. (D) Immunofluorescence analyses of pluripotent markers OCT4 in human iPSCs cultured on ET-MEF and MMC-HDF feeders. Nuclei were stained by DAPI. Scale bar, 200 μm. Student’s T test was performed with one way analysis. Data indicate mean ± SD, **P < 0.01, n =3.

Figure S2. Analysis of the collagen genes of HDF cells treated with ethanol.


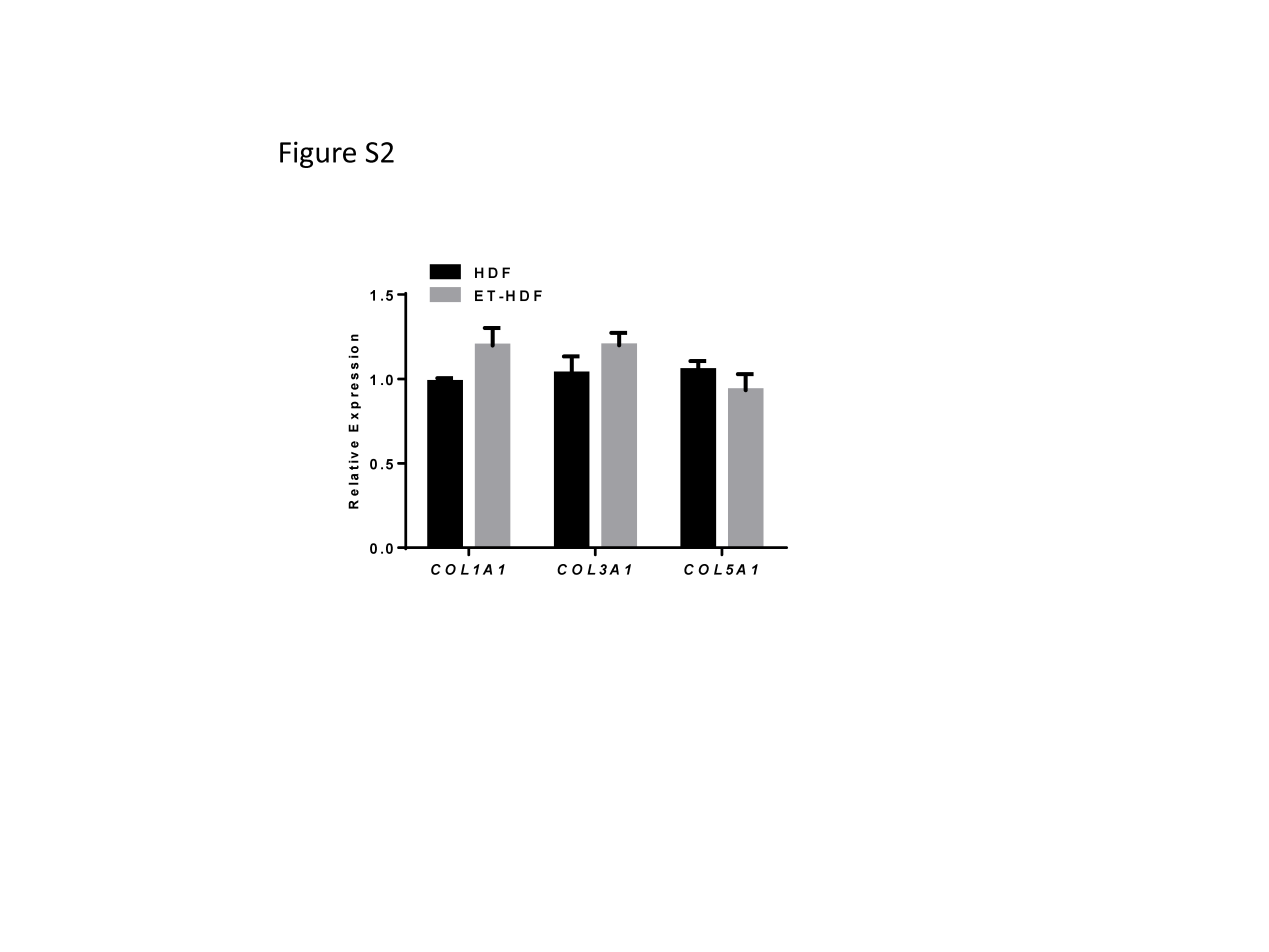


Figure S2. qRT-PCR analysis of detect the collagen genes *COL1A1, COL3A1, COL5A1* of HDF cells treated with ethanol. Data indicate mean ± SD, n =3.
